# Association of intrapatient tacrolimus variability and concentration-to-dose ratio with outcomes in pediatric kidney transplantation

**DOI:** 10.1007/s00467-025-06872-5

**Published:** 2025-07-21

**Authors:** Maral Baghai Arassi, Nora Fisch, Manuel Feißt, Kai Krupka, Britta Höcker, Alexander Fichtner, Nele Kanzelmeyer, Jens König, Anette Melk, Jun Oh, Lars Pape, Lutz T. Weber, Marcus Weitz, Burkhard Tönshoff

**Affiliations:** 1 Department of Pediatrics I, Medical Faculty, University Children‘s Hospital, Heidelberg University, Im Neuenheimer Feld 430, 69120 Heidelberg, Germany; 2 Molecular Systems Biology Unit, European Molecular Biology Laboratory (EMBL), Heidelberg, Germany; 3 Institute of Medical Biometry, Heidelberg University, Heidelberg, Germany; 4 Department of Pediatric Kidney, Liver and Metabolic Diseases and Neuropediatrics, Hannover Medical School, Hannover, Germany; 5 Department of General Pediatrics, University Children’s Hospital Münster, Münster, Germany; 6 Department of Pediatric Nephrology, University Children’s Hospital, University Medical Center Hamburg-Eppendorf, Hamburg, Germany; 7 Clinic for Paediatrics III, Essen University Hospital, Essen, Germany; 8 Pediatric Nephrology, Faculty of Medicine, Children’s and Adolescents’ Hospital, University Hospital of Cologne, University of Cologne, Cologne, Germany; 9 Department of General Pediatrics and Hematology/Oncology, University Children’s Hospital, University Hospital Tübingen, Tübingen, Germany

**Keywords:** Pediatric kidney transplantation, Tacrolimus, Intrapatient variability, Concentration-to-dose ratio, Allograft rejection

## Abstract

**Background:**

Data on the relevance of tacrolimus intrapatient variability (TacIPV) and concentration-to-dose ratio (C/D ratio) as an approximation of tacrolimus metabolism for predicting outcomes in pediatric kidney transplant (pKTx) recipients are scarce.

**Methods:**

We conducted a multicenter retrospective study of 255 pKTx recipients from the CERTAIN registry. TacIPV was quantified as the coefficient of variation (CV%) during months 6–12 post-transplant. In addition, the C/D ratio, corrected for body surface area, was calculated for the first 6 months post-transplant. Cutoffs were determined by minimization of log-rank *P* values: 23% for TacIPV and 1.0 for C/D ratio. Rejection episodes were classified according to the Banff criteria in the period following marker quantification.

**Results:**

A total of 13,159 tacrolimus trough blood levels were analyzed, with a median of 52 (IQR, 41–63) measurements per patient. High TacIPV (> 23%) during months 6–12 post-transplant was associated with an increased risk of rejection beyond 12 months post-transplant (hazard ratio (HR) 1.04, 95% CI 1.01–1.06, *P* = 0.002; Kaplan–Meier analysis *P* = 0.002). Similarly, a low C/D ratio (< 1.0), i.e., rapid tacrolimus metabolism, during the first 6 months was associated with a higher risk of rejection between months 6 and 12 (inverse HR 3.13, 95% CI 1.01–9.09, *P* = 0.04; Kaplan–Meier analysis *P* = 0.011).

**Conclusions:**

This largest to date multicenter study determines pediatric-specific cutoff values for TacIPV and tacrolimus C/D ratio as a predictive marker for graft rejection. Patients with these risk factors should be closely monitored and their immunosuppressive therapy adjusted accordingly.

**Graphical Abstract:**

**Figure:**
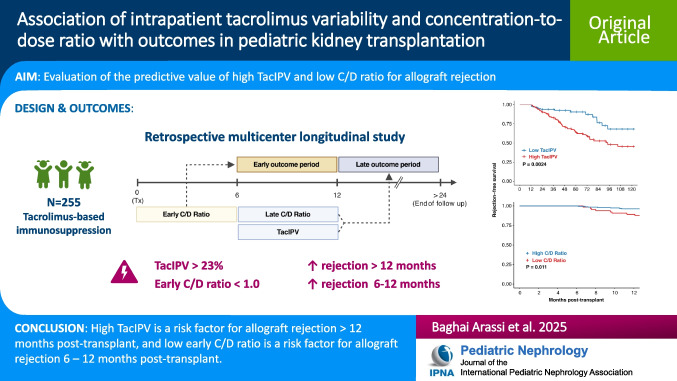
A higher resolution version of the Graphical abstract is available as Supplementary information

**Supplementary Information:**

The online version contains supplementary material available at 10.1007/s00467-025-06872-5.

## Introduction

Kidney transplantation (KTx) is the treatment of choice for children with kidney failure. Advances in immunosuppressive therapy, particularly the use of calcineurin inhibitors (CNI) such as tacrolimus and the antimetabolite mycophenolate mofetil (MMF), along with anti-infective prophylaxis, have led to significant improvements in short-term outcomes. Nevertheless, long-term graft survival remains suboptimal [1]. Although CNI-induced nephrotoxicity was traditionally viewed as the primary cause of late graft failure, recent evidence points to a multifactorial and more complex etiology [2, 3]. Among these factors, allograft rejection plays a critical role as the leading cause of graft loss [4]. Although several risk factors for allograft rejection have been identified [5], there is a lack of non-invasive predictive markers to reliably identify patients at risk. Recently, high intrapatient variability of tacrolimus trough blood levels (TacIPV) [6–8] and low concentration-to-dose (C/D) ratio [9–11] have been proposed as potential predictive markers.

Tacrolimus remains a key component of contemporary immunosuppressive regimens; nonetheless, its narrow therapeutic window and significant inter- and intra-individual variability present ongoing challenges. TacIPV reflects fluctuations in tacrolimus trough blood concentrations over time and is influenced by multiple factors such as diet, gastrointestinal disturbances, pharmacogenetics, drug interactions, drug formulation, and medication adherence [8]. The C/D ratio, on the other hand, serves as a surrogate marker of tacrolimus metabolism, calculated by dividing the trough blood concentration by the corresponding daily dose [10]. A low C/D ratio indicates higher tacrolimus clearance. Although direct causal evidence is limited, it is widely accepted that both variable tacrolimus exposure and high tacrolimus clearance contribute to periods of under- or overexposure, leading to immune activation and nephrotoxicity that adversely affect graft function [6, 8, 12].

In adult kidney transplant recipients, high TacIPV and low C/D ratio have been consistently associated with allograft rejection and poorer graft survival [10, 12–14]. However, comparable studies in pediatric kidney transplantation (pKTx) remain limited, in part due to the relatively small number of pKTx recipients. Pediatric patients present unique challenges in this context due to their distinct metabolic profiles, which often differ significantly from those of adults [15, 16]. Consequently, there is a critical need to establish pediatric-specific data for these predictive markers to identify patients at risk for suboptimal tacrolimus exposure and treatment failure. This is particularly important given their longer expected lifespan and the importance of ensuring long-term graft survival.

In this study, we analyzed data from the CERTAIN registry to evaluate the predictive value of TacIPV and C/D ratio for allograft rejection both during and after the first year post-transplant in 255 children at seven centers in Germany.

## Methods

### Study design and patient population

This multicenter, retrospective, longitudinal study analyzed data obtained from the CERTAIN database, including individuals who received kidney transplants between 2005 and 2021. The CERTAIN registry provides detailed profiling of defined patient groups by systematically collecting extensive longitudinal data, coupled with stringent procedures for data validation to ensure accuracy (http://www.certain-registry.eu/). Data are collected at defined intervals: baseline (before transplantation), months 1, 3, 6, 9, and 12, and subsequently at 6-month intervals. The flexible structure of the registry allows for continuous documentation of relevant data, including laboratory values and medication details, using specific case report forms designed for peri- and post-transplant follow-up.

The registry consists of two datasets: the mandatory minimum dataset, which is required for all centers, and the extended dataset, which provides deeper clinical insights by collecting additional predefined and center-specific data. Regarding baseline and outcome data, only the minimum dataset was used to maximize patient enrollment without relying on specific electronic case report forms. For this specific study, all tacrolimus trough levels and corresponding drug doses were retrieved from the medical records of the seven German study centers (Supplementary Table S1) and manually entered into the extended dataset after checking for medical plausibility according to a pre-defined study specific protocol (Supplementary Methods). The overall immunosuppressive load was assessed by the semiquantitative Vasudev score [17], modified for pediatric patients, as previously described [18].

The CERTAIN web platform (http://www.certain-registry.eu/RegApp) incorporates both automated and manual processes for data validation. During data entry, records are subjected to automatic verification against predefined plausibility criteria, and a manual quality assurance process includes local site approval and random checks by a central data quality manager. Only data that passes these checks is included in the research database. The system is accessible via a standard web browser and internet connection. Ethics committee approval was obtained from all participating centers, with the primary approval granted by the Ethics Committee of the Medical Faculty of the University of Heidelberg (approval number: S-388/2010). Informed consent was obtained from parents or legal guardians and, when appropriate, directly from the patient. All study procedures, including the administration of immunosuppressive regimens, were conducted according to local institutional guidelines. Routine follow-up included the collection of anthropometric, clinical, and biochemical parameters. The study was conducted in accordance with the principles of the Declaration of Helsinki and the Istanbul Declaration on Organ Trafficking and Transplant Tourism and adhered to the STROBE guidelines (https://www.strobe-statement.org/; Supplementary Methods).

### Inclusion and exclusion criteria

**Fig. 1:**
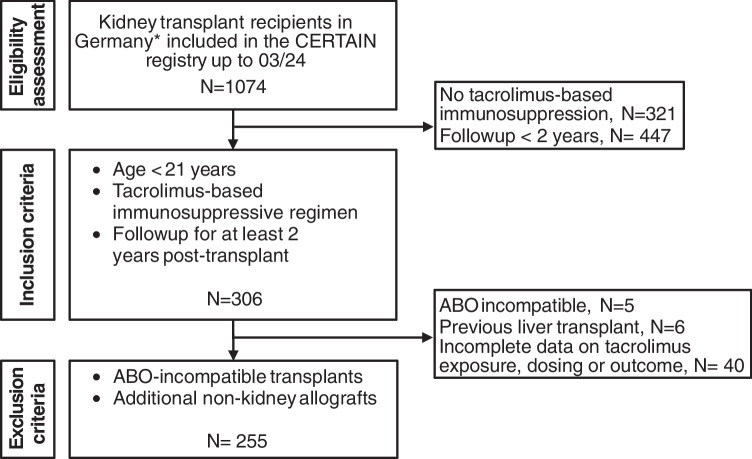
Flow diagram of patient inclusion and exclusion. Patient flow diagram showing the inclusion and exclusion criteria applied to the CERTAIN registry data to generate the cohort for the current study. Asterisk (*) indicates German centers included: Cologne, Essen, Hamburg, Hannover, Heidelberg, Münster, Tübingen

The CERTAIN registry is open to all patients younger than 21 years who are undergoing kidney transplantation, provided that informed consent is obtained from caregivers and/or the patients themselves when appropriate. No exclusion criteria specific to the registry were applied, ensuring wide eligibility and a representative transplant population. For this study, we included kidney allograft recipients in Germany who were maintained on a tacrolimus-based immunosuppressive regimen. Eligibility for analysis required a complete minimum dataset, including at least 2 years of post-transplant follow-up. Recipients of ABO-incompatible kidney transplants or those who underwent transplantation of additional non-kidney allografts were excluded. An overview of the inclusion and exclusion criteria is presented in Fig. 1.

### Outcome events

**Fig. 2:**
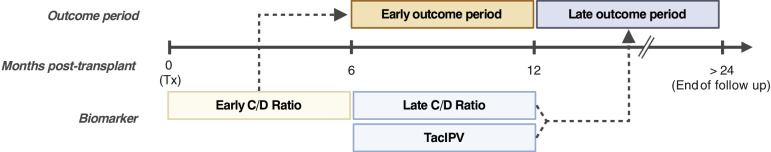
Schematic overview of tacrolimus quantification and outcome periods. The thick horizontal arrow represents the post-transplant follow-up period in months. Above the arrow are the outcome periods, divided into an early outcome period (6–12 months post-transplant, dark yellow box) and a late outcome period (> 12 months post-transplant, dark blue box). Below the arrow are the quantification periods for the predictive markers TacIPV and C/D ratio. The C/D ratio is quantified as early (≤ 6 months post-transplant, light yellow box) and late (6–12 months post-transplant, light blue box). TacIPV, due to its high variability in the early post-transplant period, is quantified only during the 6–12 months post-transplant period (light blue box). Dashed arrows indicate which markers were used to predict events in each outcome period. Only markers measured prior to each outcome period were included in the predictions

Study outcomes included allograft rejection, opportunistic infections, and graft dysfunction. Given the low incidence of graft loss in pediatric kidney transplant recipients, we utilized a death-censored composite outcome termed allograft dysfunction, defined as either graft loss, an estimated glomerular filtration rate (eGFR) below 30 mL/min/1.73 m^2^, or a decline of more than 50% from the baseline eGFR at month 3 post-transplant, without recovery above these thresholds during follow-up. eGFR values exceeding 120 mL/min per 1.73 m^2^ at 3 months post-transplant were capped at 120 mL/min per 1.73 m^2^. eGFR was estimated using the modified Schwartz formula for patients under 18 years of age and the CKD-EPI formula for those aged 18 years and older. Rejection episodes were defined by biopsy confirmation and administration of antirejection treatment, independent of whether the biopsy was performed for surveillance or for indication (for cause). Reasons for indication biopsies were an unexplained rise in serum creatinine of more than 20% above baseline and/or the onset of persistent proteinuria exceeding 100 mg protein/mmol creatinine. All biopsies were assessed by local pathologists at the respective transplant centers. Further classification was based on graft histopathology according to the applicable Banff classification in use at the time of rejection (1997–2019). Opportunistic infections were defined as infections caused by cytomegalovirus (CMV), BK polyoma virus (BKPyV), Epstein-Barr virus (EBV), or JC virus (JCV). These infections were identified based on the treating physician's assessment using a qualitative survey to document the occurrence of the infection. Data on organ involvement were available for all documented infections. For analysis, outcome events were stratified into early (months 6–12 post-transplant) and late (> 12 months post-transplant) outcome periods (Fig. 2).

### Tacrolimus exposure and metabolism quantification

Tacrolimus exposure was evaluated based on whole blood trough concentrations recorded in the CERTAIN registry. Target trough levels were defined according to individual patient risk assessment and center-specific protocols. To calculate TacIPV, at least three separate tacrolimus trough measurements per patient were required. Due to the high degree of intra-individual variability observed within the first 6 months after transplantation, TacIPV was analyzed specifically during the 6–12-month post-transplant period. Variability was quantified by calculating the coefficient of variation (CV), defined as (standard deviation ÷ mean) × 100 and expressed as a percentage [7, 19]. To enable comparisons between CV-based TacIPV and alternative statistical methods, the mean absolute deviation (MAD) of tacrolimus trough levels was additionally calculated, using the formula MAD = {[(*X*_mean_ − *X*_1_) + (*X*_mean_ − *X*_2_) … + (*X*_mean_ − *X*_n_)] ÷ *n*} ÷ *X*_mean_, where *X* represents an individual tacrolimus trough concentration.

The tacrolimus concentration-to-dose (C/D) ratio was calculated using tacrolimus trough concentration divided by the corresponding daily dose. It was also quantified during the 6–12 month post-transplant period. In addition, its reported stability early after transplantation [10] allowed for additional quantification during the first 6 months post-transplant. Throughout this manuscript, “late C/D ratio” refers to the mean C/D ratio from 6 to 12 months post-transplant, while “early C/D ratio” refers to the mean C/D ratio within the first 6 months post-transplant. To account for body size differences in pediatric patients, the C/D ratio was adjusted for body surface area (BSA) using the formula C/D ratio_BSA_ = tacrolimus blood trough level in µg/L/[daily tacrolimus dose in mg/BSA in m^2^]. For clarity, the BSA-corrected C/D ratio (C/D ratio_BSA_) is referred to simply as C/D ratio throughout this manuscript. A schematic overview of tacrolimus quantification and outcome periods is provided in Fig. 2. Only tacrolimus measurements prior to the respective outcome periods were included for prediction of outcome events.

### Statistical analysis

Continuous variables were summarized as mean ± standard deviation or as median with interquartile range (IQR), depending on distribution, while categorical variables were reported as absolute numbers and percentages. Comparisons between high and low C/D ratio groups, as well as TacIPV subgroups, were conducted using *t*-tests for continuous data and chi-square tests for categorical variables. Time-to-event outcomes were analyzed using the Kaplan–Meier method, with comparisons between groups assessed via log-rank testing. Optimal cutoff determination was performed using log-rank *P*-value minimization. Further evaluation of time-to-event outcomes was performed using multivariable Cox proportional hazards models with forward selection. Candidate variables for inclusion in the forward selection algorithm were chosen based on clinical plausibility. Model estimates were presented along with corresponding 95% confidence intervals, including hazard ratios and inverse hazard ratios. Statistical analyses were conducted using R software (version > 4.2.0). Missing data were minimal, and no imputation procedures were applied. As this was an exploratory study, *P* values are reported descriptively without formal confirmatory interpretation. Statistical significance was defined as a *P* value less than 0.05.

## Results

### Clinical and demographic characteristics

A total of 255 pediatric kidney transplant recipients (104 girls [41%] and 151 boys [59%]) who underwent transplantation between December 2005 and January 2021 across seven transplant centers in Germany were included in this study (Table 1; Supplementary Table S1). The median age at transplantation was 11 years (IQR, 5.6–15). Most patients were of Caucasian ethnicity (96%). The leading cause of kidney disease was congenital anomalies of the kidney and urinary tract (CAKUT) (41%), followed by primary glomerular diseases (25%) and hereditary cystic or congenital disorders (15%). A deceased donor kidney was transplanted in 73% of the patients. All patients were initially treated with a combination of tacrolimus, mycophenolate mofetil (MMF), and glucocorticoids as part of their immunosuppressive regimen. Additionally, 25% of patients received induction therapy with basiliximab (17%), anti-thymocyte globulin (1%), rituximab (2%), or daclizumab (2%). The median duration of post-transplant follow-up was 60 months (IQR, 36–90).

**Table 1:** Baseline clinical and demographic characteristics stratified by TacIPV

	Total *N* = 255	Low TacIPV, *N* = 103	High TacIPV, *N* = 152	*P* value
Female recipient (*N*, %)	104 (41%)	46 (45%)	58 (38%)	0.3
Age at transplantation (median, IQR)	11 (5.6, 15)	13 (8.7, 15)	9.5 (4.2, 14)	< 0.001*
Living donor (*N*, %)	68 (27%)	27 (26%)	41 (27%)	0.893
Follow-up time in months (median, IQR)	60 (36, 90)	47 (36, 72)	66 (36, 108)	< 0.001*
Preemptive transplantation	62 (24%)	24 (23%)	38 (25%)	0.756
Primary kidney disease				0.589
CAKUT	104 (41%)	44 (43%)	60 (39%)	
Cystic/hereditary/congenital diseases	38 (15%)	13 (13%)	25 (16%)	
Primary glomerular disease	63 (25%)	22 (21%)	41 (27%)	
Secondary glomerular disease/vasculitis	21 (8%)	9 (9%)	12 (8%)	
Interstitial nephritis	6 (2%)	4 (4%)	2 (1%)	
Unknown	8 (3%)	5 (5%)	3 (2%)	
Other	15 (6%)	6 (6%)	9 (6%)	
Number of HLA mismatches (mean, SD)				
HLA-A	0.78 (0.61)	0.77 (0.58)	0.79 (0.64)	0.771
HLA-B	1.1 (0.64)	1.1 (0.62)	1 (0.64)	0.453
HLA-DR	0.87 (0.59)	0.78 (0.31)	0.93 (0.57)	0.047*
Induction therapy	65 (25%)	26 (26%)	39 (27%)	0.887
Previous kidney transplants	22 (8.6%)	12 (12%)	10 (7%)	0.278
Cold ischemia time (hours, median (IQR))				
Living donor	2.7 (2.1, 4.0)	2.7 (2.1, 3.9)	2.8 (2.2, 4)	0.41
Deceased donor	13 (10, 16)	12 (10,15)	14 (10,16)	0.363
Tacrolimus tradename				0.497
Prograf™	208 (83%)	86 (85%)	122 (82%)	
Modigraf™	42 (17%)	15 (15%)	27 (18%)	
Immunosuppressive therapy at year 1				
Tacrolimus	255 (100%)	103 (100%)	152 (100%)	
MMF	197 (77%)	87 (85%)	110 (72)	0.085
Azathioprine	18 (7%)	5 (5%)	13 (9%)	0.258
Glucocorticoids	219 (86%)	88 (85%)	131 (86%)	0.866
Everolimus	20 (8%)	6 (6%)	14 (9%)	0.324

*IQR* interquartile range, *CAKUT* congenital anomalies of the kidney and urogenital tract, *HLA* human leukocyte antigen, *hrs* hours, *MMF* mycophenolate mofetil

### Tacrolimus intrapatient variability and concentration-to-dose ratio

A total of 13,159 Tac trough concentrations with corresponding dosing information were collected, with a median of 56 (IQR 45–68) measurements per patient during the first year post-transplant. The median tacrolimus trough level was 8.4 µg/L (IQR 6.5–10.5), and the median TacIPV was 26% (IQR 19–33%). Tacrolimus trough levels remained largely stable within the period of TacIPV quantification, with no clinically meaningful difference between values at 6 months post-transplant (7.2 µg/L [IQR 5.6–8.7] and 12 months post-transplant (6.8 µg/L [IQR 5.3–8.6]).

The median early and late C/D ratios were significantly different (*P* < 0.001)_,_ with an early C/D ratio of 1.3 (IQR 1.0–1.8) and a late C/D ratio of 1.6 (IQR 1.1–2.3). There was no notable correlation between TacIPV and late C/D ratio values (Pearson’s correlation coefficient 0.04).

Baseline clinical and demographic characteristics for the high and low TacIPV and C/D ratio cohorts are represented in Table 1. No significant differences in baseline variables such as sex, induction therapy, and overall intensity of immunosuppressive therapy as assessed by the Vasudev score were observed between the TacIPV subgroups. However, the high TacIPV cohort included significantly younger patients (*P* < 0.01), with significantly longer follow-up time (*P* < 0.01). The median number of Tac trough level measurements was 10 (IQR 7–12) and 12 (IQR 9–12) in the low and high TacIPV groups, respectively.

Similarly, when stratified by C/D ratio, the cohorts were comparable with respect to baseline clinical and demographic characteristics (Table 2). However, the low C/D ratio cohort included significantly younger patients (*P* < 0.001) with a longer follow-up time (*P* = 0.029) and age-related differences in their immunosuppressive therapy at year 1 post-transplant: In the low C/D ratio cohort, more patients received Modigraf™ than Prograf™ (*P* = 0.037), and fewer patients received MMF (*P* = 0.016). The median number of tacrolimus trough level measurements was 11 (IQR 9–14) and 10 (IQR 8–12) in the low and high C/D ratio groups, respectively.

**Table 2:** Baseline clinical and demographic characteristics stratified by early C/D ratio

	Low C/D, *N* = 130	High C/D, *N* = 125	*P* value
Female recipient	53 (41%)	51 (41%)	0.996
Age at transplantation (median, IQR)	8.4 (4.1, 13)	13 (9.5, 15)	< 0.001*
Living donor	35 (27%)	33 (26%)	0.925
Follow-up time in months (median, IQR)	66 (36, 70)	54 (36, 84)	0.029*
Preemptive transplantation	32 (25%)	30 (24%)	0.909
Primary kidney disease			0.452
CAKUT	59 (45%)	45 (36%)	
Cystic/hereditary/congenital diseases	15 (12%)	23 (18%)	
Primary glomerular disease	32 (25%)	31 (25%)	
Secondary glomerular disease/vasculitis	8 (6%)	13 (10%)	
Interstitial nephritis	2 (2%)	4 (3%)	
Unknown	5 (4%)	3 (2%)	
Other	9 (7%)	3 (2%)	
Number of HLA mismatches (mean, SD)			
HLA-A	0.77 (0.62)	0.79 (0.641)	0.768
HLA-B	1.1 (0.64)	1 (0.63)	0.871
HLA-DR	0.92 (0.58)	0.82 (0.59)	0.177
Induction therapy	27 (22%)	38 (32%)	_0.062_
Previous kidney transplants	9 (7%)	13 (10%)	0.258
Cold ischemia time (hours, median (IQR))			
Living donor	2.8 (2.2, 5)	2.6 (2, 3.5)	0.071
Deceased donor	13 (10. 16)	13 (10. 15)	0.218
Tacrolimus tradename			0.037*
Prograf™	102 (78%)	111 (89%)	
Modigraf™	28 (22%)	14 (12%)	
Immunosuppressive therapy at year 1			
Tacrolimus	130 (100%)	125 (100%)	
MMF	90 (69%)	107 (86%)	0.016*
Azathioprine	11 (8%)	7 (6%)	0.372
Glucocorticoids	116 (89%)	103 (82%)	0.117
Everolimus	14 (11%)	6 (5%)	0.078

*IQR* interquartile range, CAKUT congenital anomalies of the kidney and urogenital tract, *HLA* human leukocyte antigen, hrs hours, MMF mycophenolate mofetil

### Allograft rejection and tacrolimus intrapatient variability

**Fig. 3:**
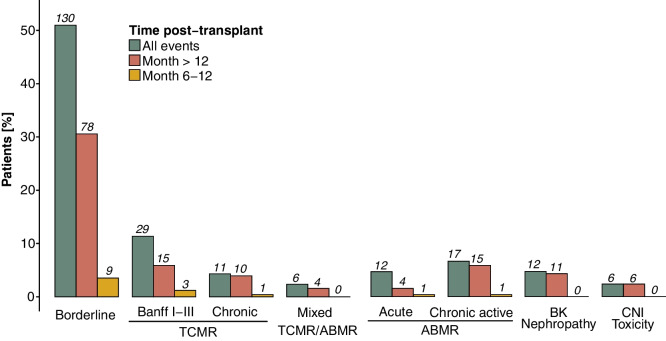
Banff-classified graft histopathology stratified by month post-transplant. Histopathology of allograft rejection graded according to Banff stratified by time post-transplant. The first event of each patient in each outcome period is considered for analysis. Color coding represents different outcome periods. ABMR, antibody-mediated rejection; BK, BK polyomavirus; TCMR, T cell-mediated rejection; CNI, calcineurin inhibitor

**Fig. 4:**
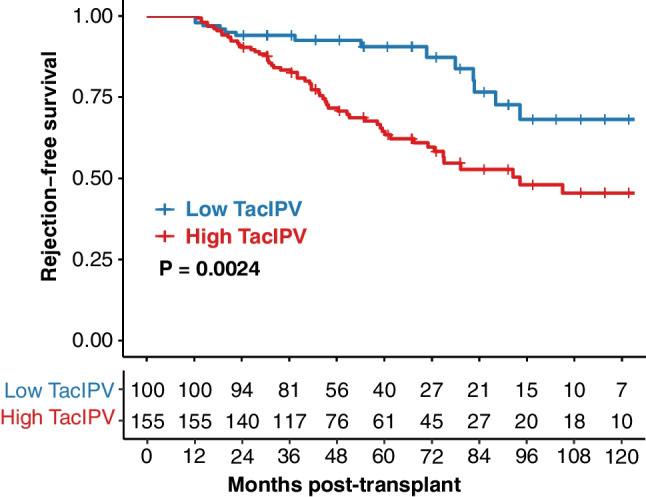
Rejection-free survival > 12 months post-transplant stratified by TacIPV. Rejection-free survival: Kaplan–Meier curves showing the probability of rejection-free survival stratified by TacIPV for the late outcome period (> 12 months post-transplant) and the follow-up period post-transplant, with high vs. low distinguished by color coding. Log-rank *P* values from the Kaplan–Meier analysis are shown to indicate the statistical significance of differences between age groups

Allograft rejection occurred in 114 patients (45%) during the entire follow-up period, with 23 patients (9%) experiencing rejection episodes within the first 6 months post-transplant, 15 patients (6%) between months 6 and 12, and 76 patients (30%) > 12 months post-transplant. The median time to a rejection episode was 31 months (IQR 4–73). Rejection episodes were stratified by outcome period (6–12 months vs. > 12 months post-transplant) and classified according to graft histopathological findings, using the Banff criteria applicable at the time of the event (1997–2019) (Fig. 3). Higher TacIPV was identified as a significant risk factor for allograft rejection > 12 months post-transplant in multivariable Cox regression analysis, with a hazard ratio of 1.04 (95% CI 1.01–1.06, *P* = 0.002) (Supplementary Table S2). Quantifying TacIPV based on Tac MAD showed comparable results with a hazard ratio of 1.45 (95% CI 1.02–2.07, *P* = 0.038). To determine the optimal cutoff for high TacIPV, log-rank *P*-value minimization showed that TacIPV values > 23% provided the best discrimination between patients who experienced allograft rejection and those who did not (Kaplan–Meier analysis, *P* = 0.003) (Fig. 4).

### Allograft rejection and concentration-to-dose ratio

Low early C/D ratio (≤ 6 months post-transplant) was identified as a significant risk factor for allograft rejection in multivariable Cox regression analysis, with an inverse hazard ratio of 3.13 (95% CI 1.05–9.09, *P* = 0.04) (Supplementary Table S2).

**Fig. 5:**
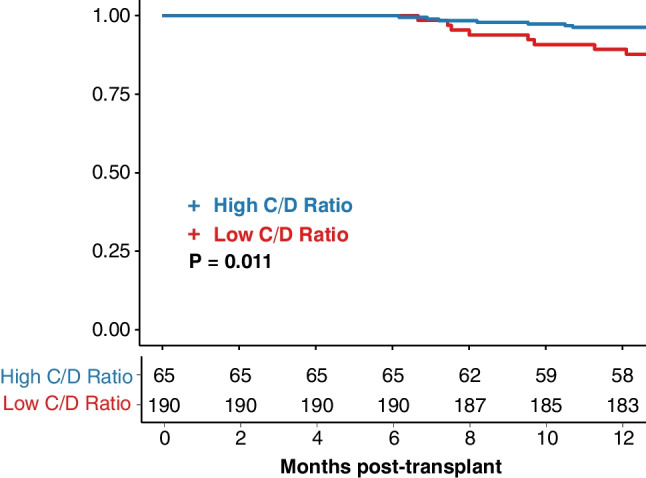
Rejection-free survival month 6–12 post-transplant stratified by C/D ratio. Rejection-free survival: Kaplan–Meier curves showing the probability of rejection-free survival stratified by C/D ratio for the early outcome period (months 6–12 post-transplant), with high vs. low distinguished by color coding. Log-rank *P* values from the Kaplan–Meier analysis are shown to indicate the statistical significance of differences between age groups

Optimal cutoff determination using log-rank *P* value minimization identified a threshold of < 1.0 as the best discriminator between patients who experienced allograft rejection and those who did not (Kaplan–Meier analysis, *P* = 0.011) (Fig. 5). In contrast, no significant association was observed between late C/D ratio (6–12 months post-transplant) and allograft rejection during the late outcome period (*P* = 0.614).

### Opportunistic infections, graft dysfunction, and other adverse events

**Fig. 6:**
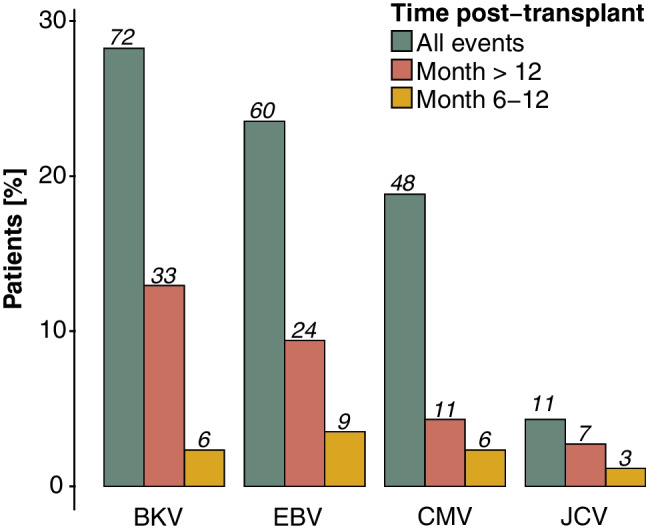
Opportunistic infections stratified by month post-transplant. Opportunistic infections stratified by the underlying virus and time post-transplant. The first event of each patient in each outcome period is considered for analysis. Color coding indicates different outcome periods. BK, BK polyomavirus; CMV, cytomegalovirus; EBV, Epstein-Barr virus; JCV, JC polyomavirus

Opportunistic infections occurred in 108 patients (42%) during the entire follow-up period, with 29 patients (11%) experiencing opportunistic infections between months 6–12 and 56 patients (22%) experiencing opportunistic infections > 12 months post-transplant. The median time to an opportunistic infection was 9 months (IQR 3–18). Opportunistic infections were categorized according to the underlying virus (Fig. 6). Although quantitative data on viral load were not available, data on organ involvement were reported. Twelve out of 72 patients with BKPyV viremia (17%) had evidence of biopsy-proven BKPyV nephropathy, 2/48 (4%) with CMV viremia had evidence of CMV gastritis/colitis or hepatitis, and 8/60 (13%) patients with EBV viremia had evidence of an upper or lower respiratory tract infection, with one patient developing an EBV-associated meningoencephalitis. There was no significant association between TacIPV, early or late C/D ratio, and opportunistic infections.

Graft dysfunction occurred in 26 patients (10%) during the entire follow-up period, with two patients (0.8%) experiencing graft dysfunction between months 6 and 12 and 24 patients (9%) experiencing graft dysfunction > 12 months post-transplant. The median time to graft dysfunction was 7 years (IQR 4–10). There was no significant association between TacIPV, early or late C/D ratio, and graft dysfunction.

The low incidence of diabetes mellitus (*N* = 9) and tremor (*N* = 2) throughout the follow-up period limited the ability to perform a meaningful statistical analysis of these events.

## Discussion

This multicenter study is the largest to date to examine the relationship between TacIPV, C/D ratio, and transplant outcomes in pediatric kidney transplant recipients. We found that high TacIPV is significantly associated with late allograft rejection occurring beyond 12 months post-transplant, while low C/D ratio is linked to earlier rejection episodes between 6 and 12 months. This is the first study to establish a pediatric-specific C/D ratio cutoff associated with rejection and the first multicenter study to demonstrate a consistent association between TacIPV and allograft rejection across pediatric transplant centers. Together, these findings suggest that TacIPV and the C/D ratio may serve as valuable non-invasive predictive markers to identify patients at risk for allograft rejection at different post-transplant times.

There is currently no consensus on the optimal statistical measures or time periods for quantifying TacIPV and C/D ratio, and standardization remains lacking. For TacIPV, coefficient of variation (CV%) is one of the most widely used approaches, particularly in pediatric transplantation studies [7, 20–28]. In our study, we found no meaningful difference between Tac CV% and Tac MAD in their association with allograft rejection. Regarding the C/D ratio, we applied BSA-correction, in line with previous work by our group [27] to ensure comparability with adult C/D ratio values. The ideal time for TacIPV and C/D ratio quantification has not been firmly established. For TacIPV, we focused on the 6- to 12-month post-transplant period [7, 8] as tacrolimus levels fluctuate substantially in the early post-transplant period, data on TacIPV beyond 12 months remain scarce, and tacrolimus trough levels in our cohort were largely stable during this interval. In contrast, previous studies, including our own [27], have assessed C/D ratio at earlier time points, as it is considered to be relatively stable soon after transplantation [10]. Nevertheless, both this study and others [29, 30] have shown that the C/D ratio increases over time. In our cohort, 23 patients shifted from the high- to the low-metabolizer group between early and late periods, with 14 of them having C/D ratios near the cutoff (0.8–1.2), indicating only marginal changes in metabolism. While the underlying mechanisms remain unclear, factors such as steroid dose reduction or complete withdrawal may influence tacrolimus metabolism, leading to changes in C/D ratio over time. This highlights the importance of considering the timing of C/D ratio assessment. The association between early C/D ratio and rejection risk was not observed with late C/D ratio, despite a higher number of events in the later follow-up period. This may reflect that early C/D ratios capture individual metabolic and immunological risk during the critical early post-transplant phase, while later rejection is likely influenced by additional factors such as immunosuppression tapering, variable adherence [26], or development of donor-specific HLA antibodies [31].

The determination of appropriate cutoff values for high TacIPV and low C/D ratio remains an area of active debate. Nonetheless, our cutoff values for both TacIPV and C/D ratio are well within previously reported ranges for both adult and pediatric kidney transplant recipients. Previously published TacIPV thresholds, derived from Tac CV% in both pediatric and adult kidney transplant studies, generally fall between 20 and 40% [21–23, 26, 32–36]. For C/D ratio, reported values range from 0.7 to 1.8 [9–12, 24, 37–42], with 1.0 being the most widely used cutoff. Cutoffs in previous studies were determined empirically, based on distribution measures (e.g., quartiles, tertiles, or median) or statistical methods. Each statistical method and time period of quantification has its strengths and limitations. Given the long and variable follow-up period in our study, we chose a cohort-independent approach based on minimization of log-rank *P* values. This method accounts for differences in follow-up time and ensures that the cutoff reflects its prognostic significance for rejection events rather than being solely arbitrarily selected.

We report the number of rejection episodes for the entire follow-up period of median 5.0 years (IQR, 3.0–7.5 years) (see Table 1), and we report all rejection types. While most studies report acute rejection rates of 10–25% within the first year post-transplant [27, 43–45], long-term data are limited. One study reported a 5-year acute rejection rate of 39% in pediatric recipients [43]. Excluding the 28 patients in our cohort who experienced chronic rejection episodes or rejection episodes after more than 5 years brings our observed rejection rate of 34% within the reported range by other investigators. In contrast, the comparatively low number of patients with graft dysfunction likely reflects the stricter definition we applied, which required a sustained eGFR < 30 mL/min/1.73 m^2^ or a > 50% decline from baseline eGFR at 3 months post-transplant. This approach was chosen to avoid capturing transient dysfunction (e.g., due to infection). eGFR values > 120 mL/min/1.73 m^2^ at 3 months were capped to control for early hyperfiltration in young recipients having received a kidney transplant from an adult donor.

Both C/D ratio and TacIPV have been shown to vary with age, with younger children typically exhibiting higher variability and lower C/D ratios. Several factors may contribute to age-related differences in tacrolimus exposure, including developmental differences in metabolism [46], more frequent dose adjustments due to infections [19, 47] (especially diarrhea, which can paradoxically increase exposure), inflammation [48], age-related variation in gut microbiota [49], and differences in tacrolimus formulation [14]. In our cohort, younger children more often received the liquid formulation, which is associated with slightly higher bioavailability. However, this does not explain their lower C/D ratios, suggesting increased tacrolimus metabolism in this age group. To account for age as a potential confounder, it was included in our multivariable Cox regression models (Supplementary Table S2). It was excluded from the final C/D ratio model by forward selection and remained in the TacIPV model without reaching statistical significance. Therefore, we found no clear evidence that age-stratified cutoffs would improve the predictive performance of these markers. Moreover, the limited number of events, particularly during early follow-up, precluded meaningful stratified cutoff determination and testing. Future studies are warranted to further explore the complex relationship between age, tacrolimus metabolism, and transplant outcomes.

Our study has several limitations. As a retrospective registry-based investigation, it does not permit direct causal relationships to be established between tacrolimus exposure, the factors influencing it (including therapy adherence) and transplant outcomes. Moreover, the limited granularity of our data hindered detailed analysis of infections such as BK polyomavirus, Epstein-Barr virus, and CMV, which were documented solely as binary variables. Nevertheless, other CERTAIN registry studies have explored post-transplant opportunistic infections in greater depth, utilizing study-specific electronic case report forms to offer a more detailed reference on this topic [18, 31, 50, 51]. Additionally, this cohort may not fully represent the broader population of kidney transplant recipients during the study period, as patients with incomplete or missing tacrolimus monitoring data were excluded. This exclusion, which may have involved patients receiving non-tacrolimus immunosuppressive regimens or those with insufficient documentation, could have contributed to an underestimation of certain clinical outcomes, such as new-onset diabetes mellitus, especially among individuals who discontinued tacrolimus due to adverse effects. In addition, as most patients in our cohort were of Caucasian descent, where CYP3A5 expressers are less common [52], the findings may not fully generalize to more diverse populations. Despite these limitations, our findings support the potential utility of TacIPV and C/D ratio as markers for identifying patients at increased risk of allograft rejection, which could guide individualized follow-up and monitoring strategies.

In conclusion, this study, the largest to date on the relevance of TacIPV and C/D ratio in pediatric kidney transplant recipients, identifies high TacIPV and low C/D ratio as significant risk factors for allograft rejection. TacIPV and C/D ratio may serve as cost-effective, non-invasive predictive markers for early identification of patients at risk for rejection. In patients with highly variable tacrolimus trough levels and low C/D ratios, potential causes of high TacIPV and increased tacrolimus metabolism should be investigated and addressed to reduce the risk of rejection and improve long-term graft outcomes.

## Supplementary information

Below is the link to the electronic supplementary material.

Graphical abstract (PPTX 355 KB)

Supplementary file2 (PDF 446 KB)

## Data Availability

The data that support the findings of this study are available from the corresponding author upon reasonable request.

## Abbreviations

ABMR: Antibody-mediated rejection
BKPyV: BK polyoma virus
BSA: Body surface area
C/D ratio: Concentration-to-dose ratio
CMV: Cytomegalovirus
CNI: Calcineurin inhibitor
CV%: Coefficient of variation in percent
EBV: Epstein-Barr virus
eGFR: Estimated glomerular filtration rate
HR: Hazard ratio
IQR: Interquartile range
JCV: JC virus
KTx: Kidney transplantation
MAD: Mean absolute deviation
MDRD: Modification of diet in renal disease
MFF: Mycophenolate mofetil
pKTX: Pediatric kidney transplantation
TacIPV: Tacrolimus intra-patient variability
TCMR: T Cell-mediated rejection
