# Supplementary material for: Association of intrapatient tacrolimus variability and concentration-to-dose ratio with outcomes in pediatric kidney transplantation
Source: Pediatr Nephrol. 2025 Jul 21;40(12):3743–54. doi: 10.1007/s00467-025-06872-5 (PMC12549751; doi:10.1007/s00467-025-06872-5)
Supplement: Supplementary file 1 — (PPTX 355 KB) [file 467_2025_6872_MOESM1_ESM.pptx]

## Slide 1
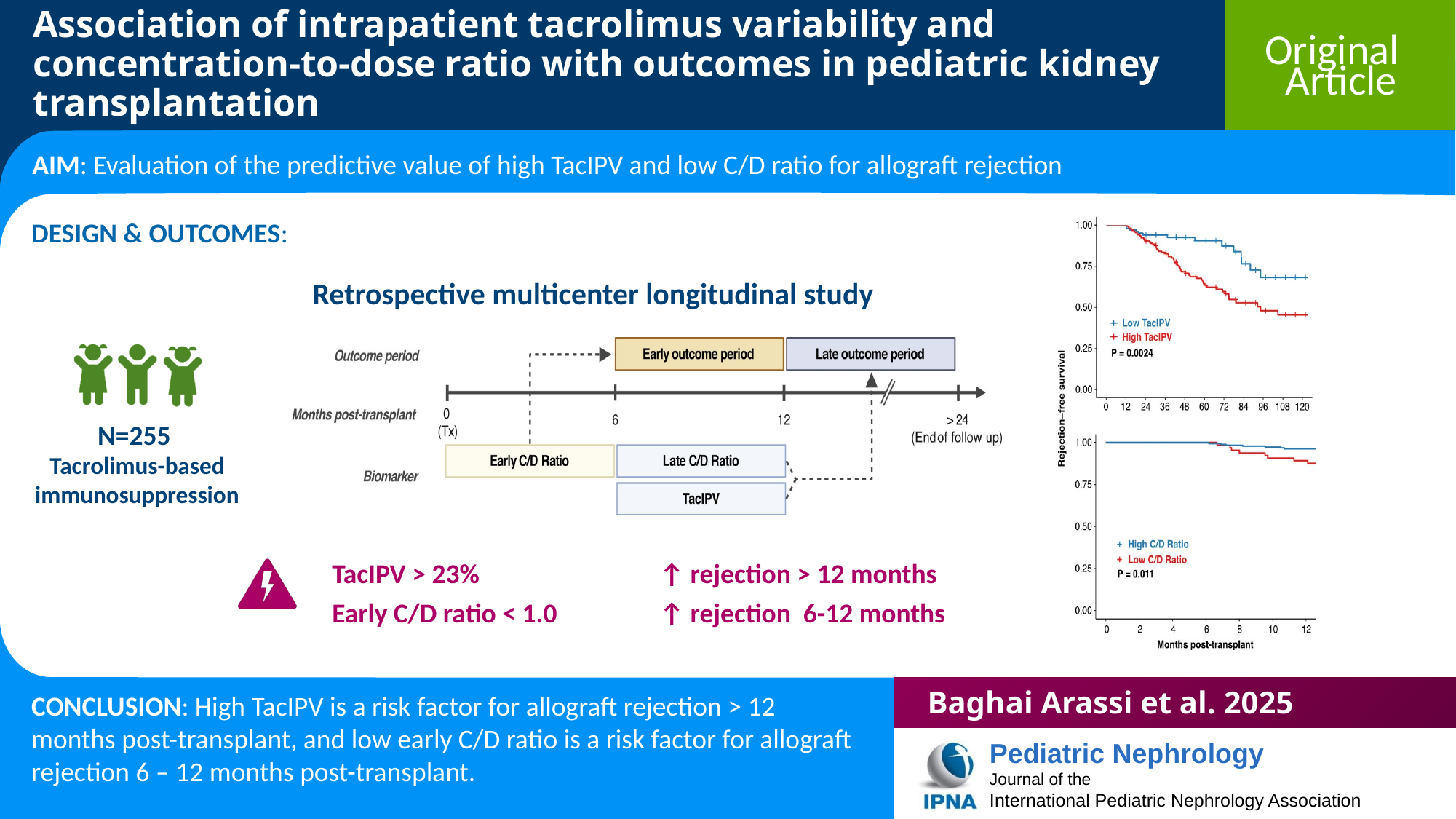

Association of intrapatient tacrolimus variability and concentration-to-dose ratio with outcomes in pediatric kidney transplantation
AIM: Evaluation of the predictive value of high TacIPV and low C/D ratio for allograft rejection
DESIGN & OUTCOMES:
Retrospective multicenter longitudinal study
N=255
Tacrolimus-based immunosuppression
TacIPV > 23% 		↑ rejection > 12 months
Early C/D ratio < 1.0	↑ rejection 6-12 months
Baghai Arassi et al. 2025
CONCLUSION: High TacIPV is a risk factor for allograft rejection > 12 months post-transplant, and low early C/D ratio is a risk factor for allograft rejection 6 – 12 months post-transplant.
