# Supplementary material for: Association of intrapatient tacrolimus variability and concentration-to-dose ratio with outcomes in pediatric kidney transplantation
Source: Pediatr Nephrol. 2025 Jul 21;40(12):3743–54. doi: 10.1007/s00467-025-06872-5 (PMC12549751; doi:10.1007/s00467-025-06872-5)
Supplement: Supplementary file 2 — Supplementary file2 (PDF 446 KB) [file 467_2025_6872_MOESM2_ESM.pdf]

Supplementary Materials

Association of intrapatient tacrolimus variability and concentration-to-dose ratio with allograft rejection, opportunistic infections and graft dysfunction in pediatric kidney transplant recipients

Maral Baghai Arassi[1,2], Nora Fisch[1], Manuel Feißt[3], Kai Krupka[1], Britta Höcker[1], Alexander Fichtner[1], Nele Kanzelmeyer[4], Jens König[5], Anette Melk[4], Jun Oh[6], Lars Pape[7], Lutz T. Weber[8], Marcus Weitz[9], Burkhard Tönshoff[1], on behalf of the CERTAIN Research Network and the ESPN working group transplantation

[1] Heidelberg University, Medical Faculty, Department of Pediatrics I, University Children's Hospital Heidelberg, Germany

[2] Molecular Systems Biology Unit, European Molecular Biology Laboratory (EMBL) Heidelberg, Germany

[3] Institute of Medical Biometry, Heidelberg University, Germany

[4] Department of Pediatric Kidney, Liver and Metabolic Diseases and Neuropediatrics, Hannover Medical School, Hannover, Germany

[5] Department of General Pediatrics, University Children's Hospital Münster, Münster, Germany

[6] Department of Pediatric Nephrology, University Children's Hospital, University Medical Center Hamburg-Eppendorf, Hamburg, Germany

[7] Clinic for Paediatrics III, Essen University Hospital, Essen, Germany.

[8] Pediatric Nephrology, Children´s and Adolescents´ Hospital, University Hospital of Cologne, Faculty of Medicine, University of Cologne, Cologne, Germany

[9] Department of General Pediatrics and Hematology/Oncology, University Children's Hospital, University Hospital Tübingen, Tübingen, Germany

Corresponding author: Maral Baghai Arassi
Department of Pediatrics I
Heidelberg University, Medical Faculty
Im Neuenheimer Feld 430
69120 Heidelberg
Germany
Email: Maral.BaghaiArassi@med.uni-heidelberg.de

Supplementary Tables

Table S1: Number of included patients per center

Study Center	Included patients (N)
Heidelberg	121
Essen	51
Hamburg	31
Tübingen	15
Münster	13
Hannover	13
Cologne	11

Table S2: Variables included in the multivariable Cox regression model after forward selection for risk factor analysis of allograft rejection

Outcome period	Variable	HR/Inverse HR (95% CI)	P value
**Month 6-12**	Early C/D Ratio	3.13 (1.01-9.09)	0.04
	Everolimus instead of MMF at month 6	5.74 (1.83-18.0)	0.003
**Month > 12**	TacIPV	1.04 (1.01-1.05)	0.002
	Age	0.98 (0.89-1.07)	0.059
	Modified Vasudev score at month 12	1.13 (1.03-1.23)	0.006
	Previous antilymphocyte antibody therapy	0.35 (0.09-1.45)	0.148

Variables included in forward selection: age, number of Tx, HLA-mismatches, alternative agent to MMF at month 6 or 12, modified Vasudev score at month 6 or 12, previous antilymphocyte antibody therapy, previous rejection episode, previous opportunistic infection. *HR,* hazard ratio; *CI,* confidence interval, HLA, human leukocyte antigen.

Supplementary Methods

Study Protocol

Association of intraindividual tacrolimus variability and concentration-to-dose ratio with allograft rejection, opportunistic infections and graft dysfunction in pediatric kidney transplant recipients

RATIONALE

Kidney transplantation (KTx) is the best treatment option for paediatric patients with end-stage kidney disease. The introduction of potent immunosuppressants such as the calcineurin inhibitor tacrolimus (Tac) have substantially improved short-term transplant outcome. However, long-term graft survival remains limited.[1] Reasons for premature graft loss are manifold and include allograft rejection, recurrence of primary glomerular disease and BK polyoma virus nephropathy.[2,3] The majority of complications occur due to inadequate immunosuppressive drug exposure. Hence, one pivotal goal of KTx research is to optimise immunosuppressive drug exposure to effectively prevent immune activation while limiting drug toxicity.

Tac is the backbone of immunosuppressive therapy in KTx. However, adequate Tac dosing is challenging. This has multiple reasons: First, Tac is a critical dose drug in which under-dosing leads to allograft rejection while over-dosing bears the risk of nephrotoxicity, neurotoxicity or glucose metabolism disorders. Second, Tac pharmacokinetics is characterised by overall low and variably bioavailability as well as large inter- and intra-patient variability of blood exposure. Known parameters influencing Tac variability include P450 CYP3A genotype, haematocrit, age, body weight, drug interactions, food intake and therapy adherence.[4,5]

Given the narrow therapeutic index and high exposure variability, close therapeutic drug monitoring is key. The current approach consists in measuring Tac pre-dose blood levels and subsequent oral dose adjustment. However, this approach is inherently flawed as drug doses are only adjusted retroactively and the wide and time-dependent variable therapeutic target ranges may not be suitable for all individuals and clinical settings. Yet, even after more than 30 years of clinical use, trough level measurements continue to be the gold standard. As similarly potent immunosuppressive agents are currently lacking, Tac will likely remain the cornerstone of immunosuppressive therapy in KTx throughout the next decade. Therefore, future research efforts should focus on optimising Tac dosing strategies by not only accounting for Tac blood through levels but also novel prognostic biomarkers indicative of off-target Tac exposure.

In this context, Tac intrapatient variability (TacIPV) is increasingly gaining focus. TacIPV is defined as fluctuations in Tac blood concentrations in an individual patient over a certain time period in which the dose was not changed.[6] Although they are several statistical means to quantify TacIPV, the coefficient of variation (CV) is most commonly used.[6,7] Tac CV is defined as standard deviation divided by mean Tac trough level. Another common statistical mean for TacIPV quantification is the Tac absolute mean deviation (MAD). The MAD is defined as the mean absolute standard deviation.[4,7,8] Regardless of the type of calculation, the underlying idea of TacIPV is that patients with high TacIPV are more frequently exposed to episodes of off-target drug concentrations than recipients with low TacIPV. These periods of sub- or supratherapeutic exposure can induce allograft rejection or lead to drug toxicity. In fact, a growing body of evidence implicates an association between TacIPV and poor graft survival, rejection episodes.[9–13]

Another recently proposed prognostic biomarker for transplant outcome is the Tac concentration/dose ratio (C/D ratio). The C/D ratio is considered to be a surrogate parameter for Tac

clearance, metabolism and an individual's P450 CYP3A genotype. It is defined as the Tac pre-dose blood concentration divided by the corresponding daily dose.[14] Recent studies suggested that a low C/D ratio (i.e., fast Tac metabolism) is associated with poor graft and patient survival and rejection episodes.[15–19]

Both TacIPV and C/D ratio are promising predictive biomarkers for the early detection of off-target Tac exposure before relevant post-transplant complications occur. Their clinical relevance has been widely studied in the adult patient population.[6,7,9–13] However, comparable studies in pediatric kidney transplantation remain limited, in part due to the relatively small number of pediatric transplant recipients. Pediatric patients, however, present unique challenges in this context due to their distinct metabolic profiles, which often differ significantly from those of adults[20,21]. Consequently, there is a critical need to establish pediatric-specific data for these biomarkers to identify patients at risk for suboptimal tacrolimus exposure and treatment failure. This is particularly important given their longer expected lifespan and the importance of ensuring long-term graft survival.

Implementing the presented study within the comprehensive CERTAIN registry addresses these issues by providing large patient numbers for high statistical power with the aim to define the clinical relevance and diagnostic thresholds of TacIPV and C/D ratio in paediatric KTx recipients. An in-depth analysis of TacIPV and C/D ratio would facilitate translation into patient care and address the unmet clinical need for reliable non-invasive predictive biomarkers for transplant outcome.

STUDY AIMS

Primary Study Objective

Evaluation of tacrolimus intrapatient variability (TacIPV) and concentration/dose ratio (C/D ratio) as predictive biomarkers for clinical outcome in paediatric KTx recipients. Clinical outcome parameters are:

- Allograft rejection
- Allograft dysfunction
- Opportunistic infections
- Clinical signs of tacrolimus over-exposure

See below for detailed definition of outcome events.

Secondary Study Objective

- Definition of potential diagnostic thresholds for TacIPV and C/D ratio

METHODS AND STUDY DESIGN

This is a retrospective multicentre longitudinal cohort study. The study population consists of KTx recipients registered in the CERTAIN database. Inclusion criteria are (i) Tac as part of the immunosuppressive maintenance regimen, (ii) complete minimum data set until at least 2 years posttransplant. Data will be collected at defined intervals: baseline, months 1, 3, 6, 9 and 12, and every six months thereafter. All available tacrolimus trough levels and corresponding drug doses will be retrieved from the medical records of the study centers and manually entered into the extended dataset after checking for medical plausibility.

Study Procedures

Patient selection and baseline medical history (Initial Visit)

All KTx patients registered in the CERTAIN database will be included in the initial screening. All patients with complete follow up until 2 years posttransplant will be included for further analysis. Patients with ABO incompatible transplants or those who received additional non-kidney allografts will be excluded. Parameters of interest are:

- Patient history
  - Age, sex, ethnicity
  - Primary renal disease
- Transplant history
  - Donor type
  - Number of previous transplantations
  - HLA-matching (A, B, DR)
  - Induction therapy
  - Preemptive transplantation
  - Cold ischemia time
  - Tacrolimus tradename
  - Immunosuppressive comedication at month 12 post-transplant
  - Vasudev score modified for paediatric patients at month 6 and 12 post-transplant

Transplant outcome parameters and stratification (all visits > month 1 post-transplant)

- Transplant outcome stratification (Figure 1):
  - Early transplant outcome (month 6-12 post-transplant)
  - Late transplant outcome (> month 12 post-transplant)
- Transplant outcome parameter:
  - Rejection episodes confirmed by biopsy and receiving antirejection therapy, categorized based on graft histopathology using the most recent Banff classification in effect at the time of rejection
  - Transplant-specific viral infections (BKPyV-, CMV-, EBV-DNAemia)
  - Allograft dysfunction defined as ≥50% decrease of baseline eGFR at 3 months posttransplant and/or eGFR < 30 ml/min*1.73m^2 without an increase in GFR above these thresholds during subsequent study visits. eGFR values > 120 mL/min per 1.73 m^2 at 3 months post-transplant will be set at 120 mL/ min per 1.73 m^2.
  - Clinical signs of Tac over-exposure as defined by development of glucose metabolism disorders or tacrolimus-associated tremor

Quantification of TacIPV (6-12 months post-transplant)

Data on Tac whole-blood trough levels is collected from the CERTAIN database. TacIPV is correlated to the late transplant outcome only (Figure 1).

TacIPV is calculated using the following statistical means:

- **Coefficient of variation (CV)**: CV_{Obs} = *(standard deviation ÷ mean)*100* and expressed as percentage
- **Mean absolute deviation (MAD)**: $MAD_{Obs} = \{[(X_{mean}-X_1) + \{[(X_{mean}-X_2) ...+ \{[(X_{mean}-X_n)] \div n\} \div X_{mean}$, where *X* is the Tac blood trough level

Quantification of C/D ratio (≤ 12 months post-transplant)

Data on Tac whole-blood trough levels and daily Tac dose is collected from the CERTAIN database. C/D ratio is stratified into

- **Early C/D ratio** (mean C/D ratio ≤ month *6*), which is correlated with early transplant outcome (month 6-12) and
- **Late C/D ratio** (mean C/D ratio month 6-12), which is correlated to late transplant outcome (> year 1) (Figure 1).

The C/D ratio is calculated using the following formula:

- **Body surface area-corrected C/D ratio**: *C/D ratio*$_{BSA}$ *= Tac blood trough level [ng/ml] ÷ (daily Tac dose [mg]/body surface area [m*^{2}*)])*

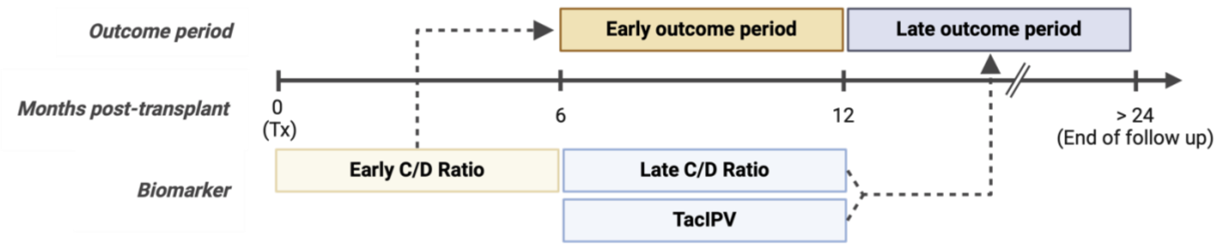

Figure 1: Time period stratification for outcome and biomarker quantification. The early C/D ratio will be correlated to the early outcome period (yellow). The late C/D ratio and TacIPV will be correlated to the late outcome period (blue).

Statistical design

Continuous variables will be summarized using either mean and standard deviation or median and interquartile range, as appropriate. Categorical variables will be described using absolute and relative frequencies. Comparisons between subgroups defined by high and low C/D ratio and TacIPV will be conducted using t-tests for continuous variables and chi-squared tests for categorical variables. Time-to-event outcomes will be analyzed using Kaplan-Meier estimators, with comparisons made using log-rank tests. For multivariable analysis of time-to-event outcomes, Cox proportional hazards regression with forward selection will be applied, with variables selected based on medical plausibility. Model estimates will be presented with 95% confidence intervals for each measure, such as hazard ratios and inverse hazard ratios. Cut-offs for TacIPV and the C/D ratio will be determined by minimizing the log-rank P values. A P value of <0.05 will be considered statistically significant.

RELEVANCE

Improving long-term patient and graft survival remains one of the major challenges in paediatric KTx medicine. The medical need for reliable non-invasive predictive biomarkers for transplant outcome is especially high in the paediatric setting, as children depend even more on a functioning graft due to their longer life expectancy. However, biomarker testing and validation is especially challenging in this cohort, as it is highly heterogeneous and patient numbers are limited. Investigating promising risk stratification tools such as TacIPV and C/D ratio in this large multicentre setting addresses these challenges by allowing high statistical power. Evaluating TacIPV and C/D ratio in this large paediatric patient cohort facilitates the implementation of these potential biomarkers in the clinical setting and potentially improves transplant outcome by allowing early identification of patients at risk for pre-

mature graft loss and proactive immunosuppressive therapy modifications. If proven useful, TacIPV and C/D ratio bear the promise of a potential paradigm shift in the therapeutic drug monitoring of tacrolimus.

References

1. Gondos A, Döhler B, Brenner H, Opelz G. Kidney graft survival in Europe and the United States: strikingly different long-term outcomes. *Transplantation*. 2013;95(2):267-274. doi:10.1097/TP.0b013e3182708ea8

2. Neuberger JM, Bechstein WO, Kuypers DRJ, et al. Practical Recommendations for Long-term Management of Modifiable Risks in Kidney and Liver Transplant Recipients: A Guidance Report and Clinical Checklist by the Consensus on Managing Modifiable Risk in Transplantation (COMMIT) Group. *Transplantation*. 2017;101(4S Suppl 2):S1-S56. doi:10.1097/TP.0000000000001651

3. Sellarés J, de Freitas DG, Mengel M, et al. Understanding the causes of kidney transplant failure: the dominant role of antibody-mediated rejection and nonadherence. *Am J Transplant Off J Am Soc Transplant Am Soc Transpl Surg*. 2012;12(2):388-399. doi:10.1111/j.1600-6143.2011.03840.x

4. Shuker N, van Gelder T, Hesselink DA. Intra-patient variability in tacrolimus exposure: Causes, consequences for clinical management. *Transplant Rev*. 2015;29(2):78-84. doi:10.1016/j.trre.2015.01.002

5. Andrews LM, Li Y, De Winter BCM, et al. Pharmacokinetic considerations related to therapeutic drug monitoring of tacrolimus in kidney transplant patients. *Expert Opin Drug Metab Toxicol*. 2017;13(12):1225-1236. doi:10.1080/17425255.2017.1395413

6. Kuypers DRJ. Intrapatient Variability of Tacrolimus Exposure in Solid Organ Transplantation: A Novel Marker for Clinical Outcome. *Clin Pharmacol Ther*. 2020;107(2):347-358. doi:10.1002/cpt.1618

7. Gonzales HM, McGillicuddy JW, Rohan V, et al. A comprehensive review of the impact of tacrolimus intrapatient variability on clinical outcomes in kidney transplantation. *Am J Transplant*. 2020;20(8):1969-1983. doi:10.1111/ajt.16002

8. Abu Bakar K, Mohamad NA, Hodi Z, et al. Defining a threshold for tacrolimus intra-patient variability associated with late acute cellular rejection in paediatric kidney transplant recipients. *Pediatr Nephrol*. 2019;34(12):2557-2562. doi:10.1007/s00467-019-04346-z

9. Borra LCP, Roodnat JI, Kal JA, Mathot RAA, Weimar W, van Gelder T. High within-patient variability in the clearance of tacrolimus is a risk factor for poor long-term outcome after kidney transplantation. *Nephrol Dial Transplant Off Publ Eur Dial Transpl Assoc - Eur Ren Assoc*. 2010;25(8):2757-2763. doi:10.1093/ndt/gfq096

10. Sapir-Pichhadze R, Wang Y, Famure O, Li Y, Kim SJ. Time-dependent variability in tacrolimus trough blood levels is a risk factor for late kidney transplant failure. *Kidney Int*. 2014;85(6):1404-1411. doi:10.1038/ki.2013.465

11. Rodrigo E, Segundo DS, Fernández-Fresnedo G, et al. Within-Patient Variability in Tacrolimus Blood Levels Predicts Kidney Graft Loss and Donor-Specific Antibody Development. *Transplantation*. 2016;100(11):2479-2485. doi:10.1097/TP.0000000000001040

12. Sablik KA, Clahsen-van Groningen MC, Hesselink DA, van Gelder T, Betjes MGH. Tacrolimus intra-patient variability is not associated with chronic active antibody mediated rejection. *PloS One*. 2018;13(5):e0196552. doi:10.1371/journal.pone.0196552

13. Vanhove T, Vermeulen T, Annaert P, Lerut E, Kuypers DRJ. High Intrapatient Variability of Tacrolimus Concentrations Predicts Accelerated Progression of Chronic Histologic Lesions in Renal Recipients. *Am J Transplant Off J Am Soc Transplant Am Soc Transpl Surg*. 2016;16(10):2954-2963. doi:10.1111/ajt.13803

14. van Gelder T, Meziyerh S, Swen JJ, de Vries APJ, Moes DJAR. The Clinical Impact of the C0/D Ratio and the CYP3A5 Genotype on Outcome in Tacrolimus Treated Kidney Transplant Recipients. *Front Pharmacol*. 2020;11:1142. doi:10.3389/fphar.2020.01142

15. Thölking G, Fortmann C, Koch R, et al. The Tacrolimus Metabolism Rate Influences Renal Function after Kidney Transplantation. Bueno V, ed. *PLoS ONE*. 2014;9(10):e111128. doi:10.1371/journal.pone.0111128

16. Schütte-Nütgen, Thölking, Steinke, et al. Fast Tac Metabolizers at Risk – It is Time for a C/D Ratio Calculation. *J Clin Med*. 2019;8(5):587. doi:10.3390/jcm8050587

17. Jouve T, Fonrose X, Noble J, et al. The TOMATO Study (Tacrolimus Metabolization in Kidney Transplantation): Impact of the Concentration–Dose Ratio on Death-censored Graft Survival. *Transplantation*. 2020;104(6):1263-1271. doi:10.1097/TP.0000000000002920

18. Nowicka M, Górska M, Nowicka Z, et al. Tacrolimus: Influence of the Posttransplant Concentration/Dose Ratio on Kidney Graft Function in a Two-Year Follow-Up. *Kidney Blood Press Res*. 2019;44(5):1075-1088. doi:10.1159/000502290

19. Bardou FN, Guillaud O, Erard-Poinsot D, et al. Tacrolimus exposure after liver transplantation for alcohol-related liver disease: Impact on complications. *Transpl Immunol*. 2019;56:101227. doi:10.1016/j.trim.2019.101227
20. Matalová P, Urbánek K, Anzenbacher P. Specific features of pharmacokinetics in children. *Drug Metab Rev*. 2016;48(1):70-79. doi:10.3109/03602532.2015.1135941
21. Batchelor HK, Marriott JF. Paediatric pharmacokinetics: key considerations. *Br J Clin Pharmacol*. 2015;79(3):395-404. doi:10.1111/bcp.12267

STROBE Statement—checklist of items that should be included in reports of observational studies

	Item No.	Recommendation	Page No.	Relevant text from manuscript
**Title and abstract**	1	(*a*) Indicate the study's design with a commonly used term in the title or the abstract	5	Line 91-96
		(*b*) Provide in the abstract an informative and balanced summary of what was done and what was found	5	Line 88-105
**Introduction**				
Background/rationale	2	Explain the scientific background and rationale for the investigation being reported	6-7	Line 111-142
Objectives	3	State specific objectives, including any prespecified hypotheses	6-7	Line 132-142
**Methods**				
Study design	4	Present key elements of study design early in the paper	7	Line 145-153
Setting	5	Describe the setting, locations, and relevant dates, including periods of recruitment, exposure, follow-up, and data collection	7-8	Line 145-174
Participants	6	(*a*) *Cohort study*—Give the eligibility criteria, and the sources and methods of selection of participants. Describe methods of follow-up<br>*Case-control study*—Give the eligibility criteria, and the sources and methods of case ascertainment and control selection. Give the rationale for the choice of cases and controls<br>*Cross-sectional study*—Give the eligibility criteria, and the sources and methods of selection of participants	6-7	Line 119-129
		(*b*) *Cohort study*—For matched studies, give matching criteria and number of exposed and unexposed<br>*Case-control study*—For matched studies, give matching criteria and the number of controls per case	NA	
Variables	7	Clearly define all outcomes, exposures, predictors, potential confounders, and effect modifiers. Give diagnostic criteria, if applicable	9-10	Line 184-221
Data sources/ measurement	8*	For each variable of interest, give sources of data and details of methods of assessment (measurement). Describe comparability of assessment methods if there is more than one group	9-10	Line 184-221
Bias	9	Describe any efforts to address potential sources of bias	10-11	Line 223-235
Study size	10	Explain how the study size was arrived at	8-9	Line 176-183, Figure 1

Quantitative variables	11	Explain how quantitative variables were handled in the analyses. If applicable, describe which groupings were chosen and why	10-11	Line 223-235
Statistical methods	12	(*a*) Describe all statistical methods, including those used to control for confounding	10-11	Line 223-235
		(*b*) Describe any methods used to examine subgroups and interactions	10-11	Line 223-235
		(*c*) Explain how missing data were addressed	11	Line 232-233
		(*d*) *Cohort study*—If applicable, explain how loss to follow-up was addressed<br>*Case-control study*—If applicable, explain how matching of cases and controls was addressed<br>*Cross-sectional study*—If applicable, describe analytical methods taking account of sampling strategy	NA	
		(*e*) Describe any sensitivity analyses	NA	
**Results**				
Participants	13*	(a) Report numbers of individuals at each stage of study—eg numbers potentially eligible, examined for eligibility, confirmed eligible, included in the study, completing follow-up, and analysed	29	Figure 1
		(b) Give reasons for non-participation at each stage	29	Figure 1
		(c) Consider use of a flow diagram	29	Figure 1
Descriptive data	14*	(a) Give characteristics of study participants (eg demographic, clinical, social) and information on exposures and potential confounders	11	Line 238-248, Table 1
		(b) Indicate number of participants with missing data for each variable of interest	11	Line 232-233
		(c) *Cohort study*—Summarise follow-up time (eg, average and total amount)	29	Table 1
Outcome data	15*	*Cohort study*—Report numbers of outcome events or summary measures over time	9	Line 184-201
		Case-control study—Report numbers in each exposure category, or summary measures of exposure		
		Cross-sectional study—Report numbers of outcome events or summary measures		
Main results	16	(*a*) Give unadjusted estimates and, if applicable, confounder-adjusted estimates and their precision (eg, 95% confidence interval). Make clear which confounders were adjusted for and why they were included	34	Table S2
		(*b*) Report category boundaries when continuous variables were categorized	NA	
		(*c*) If relevant, consider translating estimates of relative risk into absolute risk for a meaningful time period	NA	

Continued on next page

Other analyses	17	Report other analyses done—eg analyses of subgroups and interactions, and sensitivity analyses	NA	
**Discussion**				
Key results	18	Summarise key results with reference to study objectives	14	Line 313-321
Limitations	19	Discuss limitations of the study, taking into account sources of potential bias or imprecision. Discuss both direction and magnitude of any potential bias	15-16	Line 347-360
Interpretation	20	Give a cautious overall interpretation of results considering objectives, limitations, multiplicity of analyses, results from similar studies, and other relevant evidence	16	Line 361-366
Generalisability	21	Discuss the generalisability (external validity) of the study results	16	Line 361-366
**Other information**				
Funding	22	Give the source of funding and the role of the funders for the present study and, if applicable, for the original study on which the present article is based	3	Line 46-53

*Give information separately for cases and controls in case-control studies and, if applicable, for exposed and unexposed groups in cohort and cross-sectional studies.

Note: An Explanation and Elaboration article discusses each checklist item and gives methodological background and published examples of transparent reporting. The STROBE checklist is best used in conjunction with this article (freely available on the Web sites of PLoS Medicine at http://www.plosmedicine.org/, Annals of Internal Medicine at http://www.annals.org/, and Epidemiology at http://www.epidem.com/). Information on the STROBE Initiative is available at www.strobe-statement.org.
